# Sex-Dependent S1P-Related Gene Expression in Brown Adipose Tissue Across Exercise and Thermal Conditions

**DOI:** 10.3390/ijms27156674

**Published:** 2026-07-27

**Authors:** Serena Ozabrahamyan, Shinsuke Nirengi, Pablo Vidal, Lisa A. Baer, Kristin I. Stanford

**Affiliations:** 1 Dorothy M. Davis Heart and Lung Research Institute, The Ohio State University Wexner Medical Center, Columbus, OH 43210, USA; serena.ozabrahamyan@osumc.edu (S.O.); shinsuke.nirengi@osumc.edu (S.N.); pablo.vidalsouza@lilly.com (P.V.); lisa.baer@osumc.edu (L.A.B.); 2 Department of Surgery, Division of General and Gastrointestinal Surgery, The Ohio State University Wexner Medical Center, Columbus, OH 43210, USA; 3 Department of Physiology and Cell Biology, The Ohio State University Wexner Medical Center, Columbus, OH 43210, USA

**Keywords:** sphingosine-1-phosphate, brown adipose tissue, S1PR2, thermogenesis, exercise, aging, sexual dimorphism, white adipose tissue, UCP1

## Abstract

Sphingosine-1-phosphate (S1P) signaling has emerged as a regulator of metabolic homeostasis, but its relationship to brown adipose tissue (BAT) adaptation across physiological conditions remains unclear. We examined how S1P-related gene expression in BAT and subcutaneous white adipose tissue (scWAT) varies with acute exercise, chronic exercise, ambient temperature, aging, sex, and UCP1 deficiency. Young and aged wild-type mice, as well as aged UCP1 knockout (UCP1KO) mice, were studied across acute or chronic exercise paradigms and thermal conditions ranging from 4 °C to 30 °C. In BAT, acute exercise was associated with lower *S1pr1*, *S1pr2*, and *Sphk1* expression in young females, whereas aged males showed lower *S1pr2* and *Sphk1* after exercise. In scWAT, exercise-associated induction of S1P-related transcripts was most evident in aged males. Female BAT also displayed broader temperature- and chronic exercise-associated differences in *S1pr2*, *Sphk1*, and β-adrenergic receptor transcripts, whereas male BAT showed fewer transcriptional changes. In UCP1KO mice, these exercise-associated patterns were altered or attenuated. Together, these findings identify a context-dependent adipose S1P-related transcriptional program that differs by sex, age, depot, ambient temperature, exercise condition, and UCP1 status.

## 1. Introduction

Brown adipose tissue (BAT) is a metabolically specialized organ that dissipates energy as heat through non-shivering thermogenesis, a process driven primarily by uncoupling protein 1 (UCP1). In contrast to white adipose tissue, BAT is enriched in mitochondria and uses UCP1 to uncouple oxidative phosphorylation, thereby converting chemical energy into heat and contributing to body temperature regulation [1]. Because BAT can increase whole-body energy expenditure, activation of BAT and browning of white adipose tissue have been linked to protection against obesity and type 2 diabetes [2].

Exercise is a major physiological stimulus that induces systemic metabolic adaptation, including remodeling of adipose tissue [3,4]. It also increases circulating lipid mediators that function as interorgan signals. Among these, sphingosine-1-phosphate (S1P) is an exercise-responsive bioactive lipid that rises in plasma following moderate-intensity exercise, in part because of increased sphingosine availability from skeletal muscle [5]. S1P is generated primarily by sphingosine kinase 1 (Sphk1) and signals through five G protein-coupled receptors (S1pr1–S1pr5) with distinct tissue distribution and downstream signaling properties [6,7]. In adipose tissue, S1PR1 promotes preadipocyte proliferation, whereas S1PR2 suppresses proliferation but promotes adipogenic differentiation [8,9]. Consistent with these roles, inhibition or genetic deletion of S1PR2 improves adipocyte hypertrophy and glucose intolerance in mouse models of obesity [8,9]. Emerging evidence also links sphingolipid metabolism directly to BAT biology. Sphingolipids, including ceramides and S1P, accumulate in aged murine BAT and impair brown adipocyte differentiation and thermogenic capacity [10]. Notably, pharmacological inhibition of S1PR2 promotes brown adipogenesis and increases *Ucp1* expression [10], suggesting that adipose S1P signaling may be a druggable axis in age-related metabolic decline. In humans, circulating S1P correlates with BAT activity after cold exposure [11], further supporting the physiological relevance of this pathway in thermogenic adipose tissue. However, whether S1P-related pathways are regulated in BAT by physiological stimuli such as exercise and ambient temperature remains poorly defined.

Beyond classical adrenergic control, BAT thermogenesis and UCP1 activity are influenced by ambient temperature, exercise training, aging, and sex [3,4,10,12,13,14,15,16]. These variables shape thermogenic demand and adipose tissue remodeling, yet their relationship to S1P-related gene expression in BAT has not been systematically examined. Defining how these physiological contexts alter the adipose S1P axis may help clarify whether this pathway contributes to adaptive remodeling in thermogenic tissues.

BAT biology and S1P signaling both shows marked sexual dimorphism. Females generally exhibit greater BAT activity and thermogenic capacity than males, and sex differences in adrenergic receptor expression may further contribute to these divergent responses [3,4,12,17]. In parallel, circulating S1P levels are higher in women than in men during reproductive age, and estrogen has been shown to stimulate S1P production and export [18,19]. Together, these observations support sex as an important biological variable in studies of adipose S1P-related signaling, but this relationship has not been examined in BAT across differing physiological conditions.

In this study, we examined how acute exercise, chronic exercise, ambient temperature, aging, sex, and UCP1 deficiency are associated with S1P-related gene expression in BAT and subcutaneous white adipose tissue (scWAT). By comparing transcriptional responses across these physiological contexts, we sought to determine whether the adipose S1P axis exhibits sex- and condition-specific regulation. These data identify a context-dependent transcriptional signature that may help guide future mechanistic studies of S1P signaling in thermogenic adipose tissue.

## 2. Results

### 2.1. Temperature-Associated Differences in S1P-Related Expression in Female BAT

**Figure 1:**
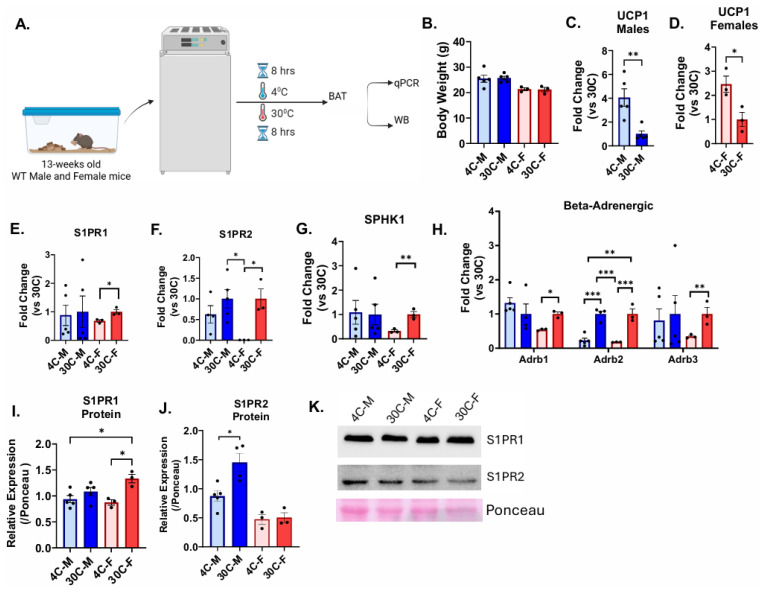
Thermal stress modulates S1P signaling and adrenergic receptor expression in murine adipose tissue. (**A**) Experimental schematic of the acute thermal exposure protocol. (**B**) Body weight of male and female mice at 4 °C and 30 °C. (**C**,**D**) Relative mRNA expression of *Ucp1* in (**C**) male and (**D**) female BAT. (**E**–**G**) Relative mRNA and protein expression of S1P signaling components in BAT. (**H**) Relative mRNA expression of β-adrenergic receptors in BAT. (**I**,**J**) Protein quantification was normalized to Ponceau staining. (**K**) Representative Western blot images. Data are presented as fold change relative to the respective 30 °C groups and expressed as mean ± SEM (n = 3–5 per group). Statistical significance in two groups within sex was determined by Student’s *t*-test. (**B**–**K**) were analyzed separately by sex. For two-condition comparisons within sex, unpaired two-tailed Student’s *t*-tests were used. Statistical significance between groups was determined by Ordinary One-way ANOVA with Tukey’s post hoc test; * *p* < 0.05, ** *p* < 0.01, *** *p* < 0.001. No additional correction was applied across distinct genes or experiments, which were analyzed independently. Blue bars: males; red bars: females. Created in BioRender. Ozabrahamyan, S. (2026) https://BioRender.com/hyn1dkw.

To compare thermal regulation of S1P-related transcription, 13-week-old wild-type male and female mice were exposed for 8 h to acute cold (4 °C) or thermoneutrality (30 °C) (Figure 1A). First, we confirmed that body weight differed by sex, with males weighing more than females across both temperature conditions but did not change within sex over the 8 h exposure period (Figure 1B). Because *Ucp1* is a cold-responsive thermogenic gene in BAT, we next measured *Ucp1* mRNA expression. As expected, acute cold exposure robustly increased *Ucp1* expression in BAT in both sexes (Figure 1C,D) [1].

When comparing thermoneutrality with acute cold exposure, female mice showed significantly higher BAT expression of *S1pr1* (Figure 1E), *S1pr2* (Figure 1F), and *Sphk1* (Figure 1G) at 30 °C than after cold exposure, whereas these transcripts did not differ significantly in males. Thermoneutrality in females was also associated with higher expression of *Adrb1*, *Adrb2*, and *Adrb3* (Figure 1H) despite lower *Ucp1* expression (Figure 1D). S1PR1 protein was significantly lower in cold-exposed females compared to thermoneutral females (Figure 1I–K), whereas males showed no significant temperature associated change. Furthermore, temperature-associated S1PR2 protein changes were only detected in cold-exposed males. Together, these findings indicate that BAT exhibits distinct temperature-associated transcriptional responses in females and males, with a broader S1P- related response evident in female BAT.

### 2.2. Acute Exercise Is Associated with Distinct S1P-Related Transcriptional Changes in BAT and scWAT in an Age- and Sex-Dependent Manner

**Figure 2:**
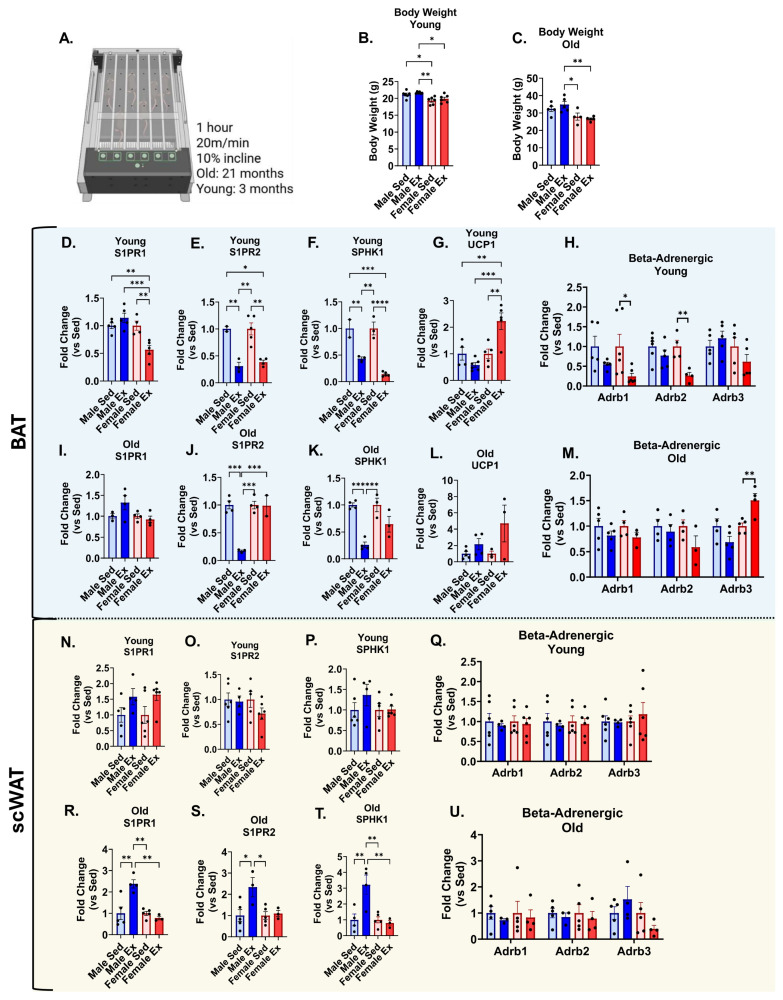
Divergent regulation of the S1P signaling pathway by acute exercise in brown and white adipose tissues is age- and sex-dependent. (**A**) Experimental schematic of the acute exercise protocol (1 h, 20 m/min, 10% incline) in young (3 months) and old (21 months) mice. (**B**,**C**) Body weight profiles of (**B**) young and (**C**) old male and female sedentary (Sed) and exercised (Ex) cohorts. (**D**–**M**) Relative mRNA expression of S1P signaling components (*S1pr1*, *S1pr2*, *Sphk1*), (**G**,**L**) thermogenic marker *Ucp1*, and (**H**,**M**) beta-adrenergic receptors (*Adrb1*, *Adrb2*, *Adrb3)* in brown adipose tissue (BAT) of young (**D**–**H**) and old (**I**–**M**) mice. (**N**–**U**) Relative mRNA expression of (**N**–**P**) S1P signaling components and (**Q**,**U**) beta-adrenergic receptors in subcutaneous white adipose tissue (scWAT) of young (**N**–**Q**) and old (**R**–**U**) mice. Data are presented as fold change relative to the respective sedentary groups and expressed as mean ± SEM (n = 3–6/group). For each panel, sedentary versus acute exercise comparisons were analyzed within age, sex, and depot using unpaired two-tailed Student’s *t*-tests. Statistical significance between groups was determined by Ordinary One-way ANOVA with Tukey’s post hoc test; * *p* < 0.05, ** *p* < 0.01, *** *p* < 0.001, **** *p* < 0.0001. Blue bars: males; red bars: females. Created in BioRender. Ozabrahamyan, S. (2026) https://BioRender.com/hyn1dkw.

We next examined whether acute exercise was associated with S1P-related gene expression in adipose tissue from young (3-month-old) and aged (21-month-old) wild-type male and female mice following a single treadmill bout (20 m/min, 10% incline, 1 h; Figure 2A). Body weight differed by sex and age across groups (Figure 2B,C). In BAT from young mice, acute exercise was associated with significantly lower *S1pr1* expression in females but not males (Figure 2D). In the same cohort, *S1pr2* and *Sphk1* were also significantly reduced after exercise, and *Ucp1* expression was significantly increased in young, exercised females (Figure 2E–G). Adrenergic receptor transcripts showed a modest decrease in *Adrb1* and *Adrb2* in young-exercised females (Figure 2H).

In BAT from aged mice, acute exercise was associated with significantly lower *S1pr2* and *Sphk1* expression in males, whereas females showed no significant changes in these transcripts (Figure 2J,K). By contrast, *S1pr1* and *Ucp1* did not differ significantly with exercise in aged mice of either sex (Figure 2I,L). Aged females showed a significant increase in *Adrb3* expression after exercise (Figure 2M).

In scWAT, young mice showed no significant exercise-associated changes in S1P-related transcripts in either sex (Figure 2N–P). In contrast, scWAT from aged male mice showed significantly higher *S1pr1*, *S1pr2*, and *Sphk1* expression after exercise, whereas aged females and young animals of both sexes showed no such responses (Figure 2R–T). Adrenergic receptor transcripts in scWAT did not differ significantly in any group (Figure 2Q,U). Together, these findings indicate that acute exercise engages adipose S1P-related transcriptional programs in a manner that depends on age, sex, and depot, with the strongest responses observed in BAT from young females and scWAT from aged males.

### 2.3. S1P-Related Expression Patterns During Long-Term Exercise Differ by Sex and Temperature

**Figure 3:**
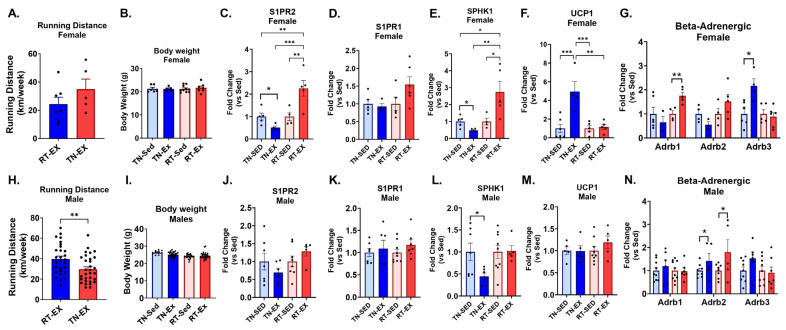
Divergent regulation of the S1P signaling pathway by 2-week wheel-cage exercise under thermoneutral and room temperature conditions. Physiological and BAT gene expression data from (**A**–**G**) female and (**H**–**N**) male mice. (**A**,**H**) Average daily running distances (km/week) for (**A**) females and (**H**) males. (**B**,**I**) Body weight profiles of (**B**) female and (**I**) male cohorts. (**C**–**F**,**J**–**M**) Relative mRNA expression of S1P signaling pathway components and the thermogenic marker *Ucp1* in (**C**–**F**) females and (**J**–**M**) males. (**G**,**N**) Relative mRNA expression of beta-adrenergic receptors in (**G**) females and (**N**) males. Data are presented as fold change relative to the respective sedentary groups and expressed as mean ± SEM (n = 4–6 per group). Running distance and bodyweight males (n = 34 per group) and females (n = 5–7 per group). Panels comparing RT-sedentary, RT-exercise, TN-sedentary, and TN-exercise groups within sex were analyzed by ordinary one-way ANOVA followed by Tukey’s multiple-comparison post hoc test; * *p* < 0.05, ** *p* < 0.01, *** *p* < 0.001. No additional correction was applied across distinct genes or experiments, which were analyzed independently. Blue bars: thermoneutrality; red bars: room temperature. Blue bars indicate thermoneutrality (TN; 30 °C) red bars indicate room temperature (RT; 22 °C) Light-shaded bars: Sedentary (Sed); Dark-shaded bars: Exercised (Ex).

To examine S1P-related transcription during chronic exercise under different thermal conditions, we measured BAT mRNA expression in 3-month-old male and female mice following 2 weeks of voluntary wheel running at room temperature (RT; 22 °C) or thermoneutrality (TN; 30 °C). Males ran significantly farther at RT than at TN, whereas females ran similar distances under both conditions (Figure 3A,H). Body weight did not differ significantly across exercise and temperature groups in either sex (Figure 3B,I).

In female mice, *S1pr2* and *Sphk1* expression was significantly higher in the RT-exercise group than in TN-sedentary controls, whereas TN-exercise was associated with significantly lower *S1pr2* and *Sphk1* relative to RT-sedentary mice (Figure 3C,E). *S1pr1* expression did not differ significantly across groups in females (Figure 3D). *Ucp1* expression was highest in TN-exercised females and was significantly lower in TN-sedentary, RT-sedentary, and RT-exercise groups relative to that condition (Figure 3F). Among adrenergic receptor transcripts, *Adrb1* was significantly higher in TN-exercised females, whereas *Adrb3* was significantly higher in RT-exercised females (Figure 3G).

In male mice, fewer transcriptional differences were observed across the same conditions. Exercise at thermoneutrality was associated with lower Sphk1 expression (Figure 3L), whereas S1pr1, S1pr2, and Ucp1 did not differ significantly across groups (Figure 3J,K,M). Among adrenergic receptors, Adrb2 was significantly higher in both exercise groups (Figure 3N). Together, these data show that BAT transcriptional responses to chronic exercise differ by sex and ambient temperature, with broader and more consistent S1P-related changes observed in female mice.

### 2.4. Exercise-Associated S1P-Related Expression Patterns Are Altered in UCP1KO Mice

**Figure 4:**
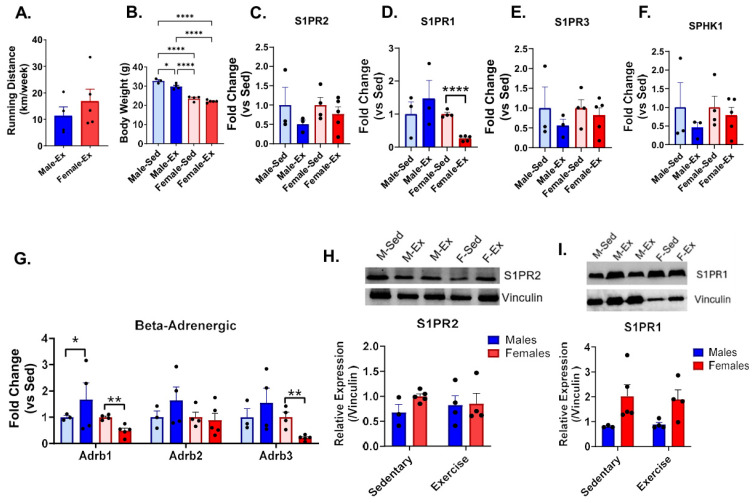
Impact of voluntary exercise on S1P signaling pathway components and beta-adrenergic receptors in UCP1-KO mice. (**A**) Average daily running distances (km/week) for male and female exercise cohorts. (**B**) Body weight profiles across male and female sedentary (Sed) and exercised (Ex) groups. (**C**–**G**) Relative mRNA expression of S1P signaling path components. (**C**–**G**) Beta-adrenergic receptors in BAT. (**H**,**I**) Representative Western blot images and corresponding densitometric protein quantification of (**H**) S1PR2 and (**I**) S1PR1 relative to Vinculin loading controls. All mice were maintained under thermoneutrality (30 °C) for 3 weeks. Data are expressed as relative expression normalized to Vinculin and presented as mean ± SEM (n = 3–5 per group). Sedentary versus exercise comparisons within sex were analyzed using unpaired two-tailed Student’s *t*-tests. Statistical significance between groups was determined by Ordinary One-way ANOVA with Tukey’s post hoc test; Exercise vs. Sed, * *p* < 0.05, ** *p* < 0.01, **** *p* < 0.0001. No additional correction was applied across distinct genes or experiments, which were analyzed independently. Blue bars: males; red bars: females. Light-shaded bars: Sedentary (Sed); Dark-shaded bars: Exercised (Ex).

To determine whether the exercise-associated transcriptional patterns observed in wild-type mice were preserved in the absence of UCP1, we evaluated 21-month-old UCP1 knockout (UCP1KO) mice after a 3-week exercise protocol at 30 °C (Figure 4). Male and female UCP1KO mice ran similar distances (Figure 4A). Body weight differed by sex, with males weighing more than females, and differed with exercise status within sex (Figure 4B).

In female UCP1KO mice, exercise was associated with significantly lower *S1pr1* expression than in sedentary controls (Figure 4D), whereas *S1pr2*, *S1pr3*, and *Sphk1* did not differ significantly between exercise and sedentary groups (Figure 4C,E,F). At the protein level, S1PR1 and S1PR2 abundance was not significantly altered by exercise in either sex (Figure 4H,I).

Adrenergic receptor transcripts showed a distinct pattern in UCP1KO mice. In exercised females, *Adrb1* and *Adrb3* were significantly lower than in sedentary females, whereas exercised males showed significantly higher *Adrb1* expression than sedentary controls (Figure 4G). Thus, the broader adrenergic receptor upregulation observed in wild-type females at thermoneutrality (Figure 3G) was not reproduced in UCP1KO mice.

Collectively, these results identify a context-dependent pattern of adipose S1P-related gene expression that varies by sex, age, depot, ambient temperature, exercise condition, and UCP1 status. The most consistent exercise-associated transcriptional differences were observed in BAT from young wild-type females and in scWAT from aged males, whereas UCP1-deficient animals exhibited a distinct and partially attenuated response profile.

## 3. Discussion

This study demonstrates that adipose S1P-related gene expression is regulated by exercise, ambient temperature, age, sex, and UCP1 status. The most pronounced differences were observed in BAT from female mice and in the altered transcriptional response of UCP1-deficient mice. These findings support a context-dependent S1P transcriptional program in adipose tissue while highlighting priorities for future mechanistic studies.

### 3.1. Sexual Dimorphism in BAT S1P-Related Transcription

A major observation across experiments was the marked sexual dimorphism in BAT transcriptional responses. Female mice showed broader induction of *S1pr1*, *S1pr2*, *Sphk1*, and several β-adrenergic receptor transcripts under thermoneutral conditions, as well as more consistent responses to acute and chronic exercise, whereas male responses were weaker or absent. This pattern aligns with prior reports of greater BAT activity, mitochondrial density, and thermogenic flexibility in females compared with males [3,4,12]. Sex differences in sympathetic tone and adrenergic receptor distribution may contribute to these divergent transcriptional programs [17].

In parallel, circulating S1P levels are higher in women than in men during reproductive age, and estrogen has been shown to stimulate S1P production and export via ABC transporters [18,19]. Estrogen receptor signaling can directly modulate sphingolipid metabolism and may enhance *Sphk1* activity in adipose tissue [18,19]. Although the present experiments were not designed to test hormone dependence, the consistency of female-biased responses across thermal, exercise, and age contexts supports sex as an important biological variable in adipose S1P-related transcriptional regulation. Future studies using ovariectomy or estrogen replacement will be necessary to establish causal roles for sex hormones in mediating these differences.

### 3.2. Temperature-Associated Regulation and the Paradox of Elevated ADRB Expression at Thermoneutrality

At thermoneutrality (30 °C), female BAT exhibited higher expression of *S1pr1*, *S1pr2*, and *Sphk1* compared with cold exposure (4 °C), despite lower *Ucp1* expression. This pattern was not observed in males. The elevation of *S1pr2* at thermoneutrality is notable because S1PR2 signaling has been linked to adipogenic differentiation and adipocyte hypertrophy [8,9]; its higher expression under conditions of reduced thermogenic demand may reflect a transcriptional state favoring lipid storage or tissue remodeling over UCP1-dependent heat production. Whether S1P signaling actively suppresses *Ucp1* expression or merely tracks with a reduced thermogenic transcriptional state remains to be determined.

Thermoneutrality in females was also associated with higher expression of *Adrb1*, *Adrb2*, and *Adrb3* despite lower *Ucp1* mRNA. While this inverse relationship appears paradoxical, a more likely explanation is that cold exposure (4 °C) triggers sustained sympathetic activation over the 8 h exposure period, which may induce β-adrenergic receptor desensitization and transcriptional downregulation as a negative feedback mechanism. At thermoneutrality (30 °C), sympathetic drive to BAT is minimal, so this activation-induced suppression does not occur, allowing ADRB transcripts to remain at higher steady-state levels. Importantly, ADRB transcript abundance does not necessarily equate to receptor activation or thermogenic output, which may explain how higher receptor mRNA can coexist with lower *Ucp1* expression. In other words, the higher ADRB expression at 30 °C may represent a basal, non-desensitized state rather than active compensatory upregulation. The observation that this pattern is most evident in females is consistent with their higher basal sympathetic tone and greater BAT adrenergic sensitivity [3,4,17].

In addition, *Adrb1* and *Adrb2* were evaluated alongside *Adrb3* because all three β-adrenergic receptor subtypes are expressed in BAT and serve distinct functions: while ADRB3 is the principal mediator of thermogenesis and lipolysis in mature brown adipocytes, ADRB1 is expressed in brown adipocyte precursors and mediates cAMP-dependent proliferation, and ADRB2 is detectable in BAT and believed to localize primarily to the vascular system, where activation promotes vasodilation and increased blood flow [20,21,22]. The coordinated expression of all three subtypes at thermoneutrality may therefore reflect a broader maintenance of adrenergic sensitivity and vascular readiness when UCP1-dependent heat production is minimally required.

### 3.3. Age- and Depot-Dependent Responses to Acute Exercise

In young mice, acute exercise was associated with lower *S1pr1*, *S1pr2*, and *Sphk1* expression in female BAT, whereas aged male BAT showed reduced *S1pr2* and *Sphk1*. These findings suggest that S1P-related transcriptional responses shift with age and differ by depot. The reduction in *S1pr2* after acute exercise in young females is particularly notable given that S1PR2 generally suppresses adipocyte proliferation and promotes differentiation [8,9]; lower *S1pr2* may therefore reflect a transient transcriptional state favoring precursor cell dynamics or reduced adipogenic signaling during the immediate post-exercise period.

By contrast, scWAT from aged males showed significant exercise-associated induction of *S1pr1*, *S1pr2*, and *Sphk1*, a response that is absent in young mice and in females. This age-dependent, depot-specific induction may reflect altered lipid handling, increased sphingosine availability, or differential sympathetic responsiveness in aged male scWAT [13,14,16]. Because we did not directly assess thermogenic activity, receptor signaling, or lipid flux, the functional significance of this shift remains uncertain. Nevertheless, the divergence between BAT and scWAT responses underscores that S1P-related pathways are not uniformly regulated across adipose depots, and that age and sex interact to shape these responses.

### 3.4. Chronic Exercise Under Different Thermal Conditions

Although these comparisons reflect the significant contrasts observed in the dataset, they also highlight how ambient temperature modifies the transcriptional response to chronic exercise. Female BAT showed higher *S1pr2* and *Sphk1* in the RT-exercise group compared with TN-sedentary controls, whereas TN-exercise was associated with lower *S1pr2* and *Sphk1* relative to RT-sedentary mice. This pattern suggests that ambient temperature modulates the direction and magnitude of exercise-induced S1P-related transcriptional changes in female BAT. In male BAT, by contrast, *S1pr1*, *S1pr2*, and *Ucp1* did not differ significantly across chronic exercise and temperature conditions, with only *Sphk1* showing a modest decrease at thermoneutrality. These data reinforce the conclusion that female BAT exhibits greater transcriptional plasticity in the S1P pathway under combined chronic metabolic and thermal stress.

### 3.5. UCP1-Dependence of Exercise-Associated S1P-Related Patterns

Exercise-associated transcriptional responses were diminished or absent in UCP1KO mice. In BAT from UCP1-deficient females, exercise reduced *S1pr1* mRNA but did not significantly alter *S1pr2*, *S1pr3*, or *Sphk1* relative to sedentary controls. Notably, S1PR1 and S1PR2 protein abundance was unchanged by exercise in UCP1KO mice despite the transcriptional difference in *S1pr1*. This discordance between mRNA and protein suggests that post-transcriptional or post-translational mechanisms, including mRNA stability, translational efficiency, or protein turnover, may buffer S1P receptor expression against transcriptional changes in the absence of UCP1. Alternatively, the time interval between exercise and tissue collection may have been insufficient to manifest protein-level changes, or compensatory feedback loops may stabilize receptor pools when thermogenic capacity is chronically impaired.

The broader attenuation of adrenergic receptor remodeling in UCP1KO mice also supports an association between intact thermogenic capacity and S1P-axis regulation. However, because UCP1 deficiency represents a lifelong genetic perturbation with potential developmental and compensatory adaptations, these data do not establish that acute UCP1 activity is directly required for S1P-associated remodeling. Rather, they identify UCP1 status as a critical contextual variable that modifies the adipose transcriptional response to exercise.

It should be noted that the exercise protocols differed between older wild-type and UCP1KO cohorts, both of which were 21 months old: wild-type mice underwent 2 weeks of ad libitum voluntary wheel running, whereas UCP1KO mice completed a 3-week protocol of scheduled 60 min/day wheel running. These differences in duration, pattern, and cumulative exercise dose may contribute to divergent transcriptional responses and should be considered when comparing results across genotypes.

### 3.6. Integration Across Physiological Contexts

Across experiments, several consistent patterns emerged that help contextualize the overall findings. First, female BAT consistently exhibited broader S1P-related transcriptional responses than male BAT, whether in response to acute cold, acute exercise, or chronic exercise under different thermal conditions. This sexual dimorphism was further emphasized by the attenuated response in UCP1KO females, suggesting that the female-biased transcriptional program is partially dependent on intact UCP1-mediated thermogenic capacity or its developmental consequences. Second, scWAT responses were most pronounced in aged males after acute exercise, highlighting a distinct, age-dependent depot-specific program that contrasts with the BAT-centric female responses. Third, thermoneutrality consistently unmasked S1P-related and adrenergic receptor expression patterns that were suppressed or absent under room-temperature or cold conditions, particularly in females. Together, these findings indicate that the adipose S1P axis is differentially regulated by physiological context in a manner that depends on the interaction of sex, age, adipose depot, ambient temperature, and thermogenic status.

### 3.7. Limitations and Future Directions

This study has several limitations. Most outcomes were measured at the mRNA level, and protein validation was limited to total S1PR1 and S1PR2 in select experiments. Circulating S1P concentrations, receptor activity, and direct functional readouts of BAT thermogenesis (e.g., respirometry, surface temperature) were not assessed. Consequently, the present data establish associations rather than causal relationships between S1P-related transcription and adipose tissue adaptation.

In addition, the UCP1KO model cannot distinguish acute loss of thermogenic function from broader developmental or compensatory effects that may independently alter S1P signaling. Future studies should employ inducible UCP1 knockdown or pharmacological UCP1 inhibition to address this limitation. Given its established roles in adipocyte hypertrophy and glucose homeostasis in WAT [8,9], the functional relevance of S1PR2 in BAT warrants direct investigation. Pharmacological blockade or adipose-specific genetic manipulation of *S1pr2* would clarify whether this receptor modulates BAT thermogenesis, adrenergic responsiveness, or exercise-induced remodeling. Caution is warranted, however, as the S1PR2 antagonist JTE-013 has been reported to exhibit off-target effects on sphingolipid metabolic enzymes; genetic validation will be essential to confirm S1PR2-specific roles [23]. Finally, the role of sex hormones in mediating the observed sexual dimorphism should be tested through gonadectomy and hormone replacement studies.

### 3.8. Conclusions

In summary, these data identify a context-dependent adipose S1P-related transcriptional program that differs by sex, age, depot, ambient temperature, exercise condition, and UCP1 status. The most consistent differences were observed in female BAT and in the altered response of UCP1-deficient mice, highlighting these settings as priorities for future mechanistic work. More broadly, the findings support adipose S1P-related signaling as a physiologically responsive pathway that may contribute to tissue adaptation across metabolic states and underscore the importance of considering sex as a biological variable in studies of adipose tissue biology.

## 4. Materials and Methods

### 4.1. Animals

All procedures were followed as approved by the IACUC at The Ohio State University. Female and male C57BL/6J wild-type (WT) mice (Charles River Laboratories) were studied at 3 months of age (young) and 21 months of age (old). For chronic exercise experiments, mice were individually housed in cages equipped with stainless-steel running wheels. Animals assigned to voluntary exercise groups were provided ad libitum access to running wheels 24 h/day, and voluntary running activity was recorded continuously using magnetic sensors to quantify individual exercise distance. Sedentary control mice were housed under the same ambient temperature conditions without wheel access.

Acute Treadmill Exercise: Acute treadmill experiments were performed in 3-month-old and 21-month-old WT mice using a motorized treadmill (Columbus Instruments). Mice were acclimated to the treadmill on 3 consecutive days prior to the exercise bout. Animals completed a single 60 min treadmill bout at room temperature (20 m/min, 10% incline), as described in the corresponding figure legend and Section 2. Mice were encouraged to run via gentle tactile stimulation or low electrical shock. The exercise bout was performed at room temperature. Total running distance was recorded for each mouse. This protocol was adapted from previously described methods [24,25].

UCP1KO Exercise Protocol: For studies of UCP1 deficiency, 21-month-old male and female UCP1 knockout (UCP1KO; Jackson Laboratory, Bar Harbor, ME, USA, strain #003124) mice were used. These mice underwent a 3-day acclimation period followed by a 3-week exercise protocol consisting of daily 60 min wheel-running sessions at thermoneutrality. Exercise protocols were adapted from previously described methods [25].

Temperature-Controlled Housing: For temperature-controlled studies, mice were housed in a diurnal incubator (Columbus Instruments) on a 12 h light–dark cycle. Ambient conditions were maintained at 30 °C for thermoneutrality (TN), 22 °C for room temperature (RT), or 4 °C for acute cold exposure for the durations specified for each experiment. At the end of each protocol, mice were anesthetized with isoflurane and euthanized by cervical dislocation. BAT and scWAT were rapidly excised, snap-frozen in liquid nitrogen and stored at −80 °C until they underwent analysis by qPCR or Western blot.

### 4.2. Quantitative Polymerase Chain Reaction (qPCR)

Total RNA was isolated from adipose tissue using TRIzol reagent (QIAGEN, Hilden, Germany, 79306) as previously described [24] and quantified using a NanoDrop One spectrophotometer (Thermo Fisher Scientific, Waltham, MA, USA). Complementary DNA (cDNA) was synthesized using the qScript cDNA Synthesis Kit (Quantabio, Beverly, MA, USA, 95047-500). Gene expression was measured by quantitative PCR using SYBR Green FastMix (Quantabio, Beverly, MA, USA, 95072-012) and normalized to β-actin. The primer sequences are listed in Table 1.

### 4.3. Western Blot

Adipose tissues were homogenized in RIPA buffer supplemented with 1× protease inhibitor (Sigma-Aldrich, St. Louis, MO, USA, A32953) using a tissue lyser, as previously described [24]. Protein concentration was determined by BCA assay (Thermo Fisher Scientific, Waltham, MA, USA, 23225) according to the manufacturer’s instructions. Twenty to twenty-five micrograms of protein was loaded per lane, separated by SDS-PAGE, and transferred to 0.22 µm PVDF membranes. Transfer efficiency was verified by Ponceau staining. Membranes were blocked for 1 h at room temperature in TBS-T (50 mmol/L Tris, 150 mmol/L NaCl, 0.05% Tween-20) containing 5% non-fat dry milk, incubated overnight at 4 °C with primary antibodies against S1PR1 (Thermo Fisher Scientific, Waltham, MA, USA 55133-1-AP, 1:200) or S1PR2 (Novus Biologicals/Bio-Techne, Centennial, CO, USA, NBP3-27900, 1:2000), and then incubated for 1 h at room temperature with HRP-conjugated anti-rabbit secondary antibody (NA934V, 1:1000). Bands were visualized using a Bio-Rad ChemiDoc system and normalized to vinculin (Cell Signaling Technology, Danvers, MA, USA, 13901S, 1:2000) or to total protein by Ponceau staining, as indicated for the corresponding blot.

### 4.4. Statistical Analysis

Data are presented as mean ± SEM and were analyzed using GraphPad Prism version 10. Fold change calculated as 2^−ΔΔCT^ [26]. Each experiment was analyzed independently. Comparisons between two groups were performed using unpaired two-tailed Student’s t-tests, whereas comparisons among more than two groups were analyzed by ordinary one-way ANOVA followed by Tukey’s multiple-comparison post hoc test. Male and female cohorts were analyzed separately unless otherwise indicated. A *p* value < 0.05 was considered statistically significant. The sample size (n) for each experiment is provided in the corresponding figure legend.

## Figures and Tables

**Table 1:** Primer sequences used for qPCR.

Gene Name	Forward Primer	Reverse Primer
S1pr1	CGCAGTTCTGAGAAGTCTCTGG	GGATGTCACAGGTCTTCGCCTT
S1pr2	TGTTGCTGGTCCTCAGACGCTA	AGTGGGCTTTGTAGAGGACAGG
S1pr3	GCTTCATCGTCTTGGAGAACCTG	CAGAGAGCCAAGTTGCCGATGA
Sphk1	GCTTCTGTGAACCACTATGCTGG	ACTGAGCACAGAATAGAGCCGC
Adrb1	GCTCTGGACTTCGGTAGATGTG	CGTCAGCAAACTCTGGTAGCGA
Adrb2	GAGCGACTACAAACCGTCACCA	TGGAAGTCCAGAACTCGCACCA
Adrb3	AGGCACAGGAATGCCACTCCAA	GCTTAGCCACAACGAACACTCG
β-actin	CATTGCTGACAGATGCAGAAGG	TGCTGGAAGGTGGACAGTGAGG
Ucp1	GCTTTGCCTCACTCAGGATTGG	CCAATGAACACTGCCACACCTC

## Data Availability

Data are available from the corresponding author upon reasonable request.

## Abbreviations

The following abbreviations are used in this manuscript:

S1P	Sphingosine-1-phosphate
S1PR1	Sphingosine-1-phosphate receptor 1
S1PR2	Sphingosine-1-phosphate receptor 2
S1PR3	Sphingosine-1-phosphate receptor 3
Sphk1	Sphingosine Kinase 1
Adrb1	β-1-adrenergic receptor
Adrb2	β-2-adrenergic receptor
Adrb3	β-3-adrenergic receptor
UCP1	Uncoupling protein 1
TN	Thermoneutrality
RT	Room temperature
BAT	Brown adipose tissue
scWAT	Subcutaneous white adipose tissue

## References

[1] Cannon B., Nedergaard J.A.N. (2004). Brown Adipose Tissue: Function and Physiological Significance. Physiol. Rev..

[2] Kajimura S., Spiegelman B.M., Seale P. (2015). Brown and Beige Fat: Physiological Roles beyond Heat Generation. Cell Metab..

[3] Rodriguez-Cuenca S., Pujol E., Justo R., Frontera M., Oliver J., Gianotti M., Roca P. (2002). Sex-dependent thermogenesis, differences in mitochondrial morphology and function, and adrenergic response in brown adipose tissue. J. Biol. Chem..

[4] Shashank C.G., Mandali R., Wankhade U.D. (2025). Hormones, heat, and health: A comprehensive review of sex-based differences in brown and beige fat biology. Biol. Sex. Differ..

[5] Baranowski M., Błachnio-Zabielska A.U., Charmas M., Helge J.W., Dela F., Książek M., Długołęcka B., Klusiewicz A., Chabowski A., Górski J. (2015). Exercise increases sphingoid base-1-phosphate levels in human blood and skeletal muscle in a time- and intensity-dependent manner. Eur. J. Appl. Physiol..

[6] Chatzikonstantinou S., Poulidou V., Arnaoutoglou M., Kazis D., Heliopoulos I., Grigoriadis N., Boziki M. (2021). Signaling through the S1P−S1PR Axis in the Gut, the Immune and the Central Nervous System in Multiple Sclerosis: Implication for Pathogenesis and Treatment. Cells.

[7] Chakrabarty S., Bui Q., Badeanlou L., Hester K., Chun J., Ruf W., Ciaraldi T.P., Samad F. (2022). S1P/S1PR3 signalling axis protects against obesity-induced metabolic dysfunction. Adipocyte.

[8] Asano M., Kajita K., Fuwa M., Kajita T., Mori I., Akahoshi N., Ishii I., Morita H. (2023). Opposing Roles of Sphingosine 1-Phosphate Receptors 1 and 2 in Fat Deposition and Glucose Tolerance in Obese Male Mice. Endocrinology.

[9] Kitada Y., Kajita K., Taguchi K., Mori I., Yamauchi M., Ikeda T., Kawashima M., Asano M., Kajita T., Ishizuka T. (2016). Blockade of Sphingosine 1-Phosphate Receptor 2 Signaling Attenuates High-Fat Diet-Induced Adipocyte Hypertrophy and Systemic Glucose Intolerance in Mice. Endocrinology.

[10] Gohlke S., Zagoriy V., Cuadros Inostroza A., Meret M., Mancini C., Japtok L., Schumacher F., Kuhlow D., Graja A., Stephanowitz H. (2019). Identification of functional lipid metabolism biomarkers of brown adipose tissue aging. Mol. Metab..

[11] Borup A., Donkin I., Boon M.R., Frydland M., Martinez-Tellez B., Loft A., Keller S.H., Kjaer A., Kjaergaard J., Hassager C. (2022). Association of apolipoprotein M and sphingosine-1-phosphate with brown adipose tissue after cold exposure in humans. Sci. Rep..

[12] Gómez-García I., Trepiana J., Fernández-Quintela A., Giralt M., Portillo M.P. (2022). Sexual Dimorphism in Brown Adipose Tissue Activation and White Adipose Tissue Browning. Int. J. Mol. Sci..

[13] Lehnig A.C., Stanford K.I. (2018). Exercise-induced adaptations to white and brown adipose tissue. J. Exp. Biol..

[14] Vidal P., Stanford K.I. (2020). Exercise-Induced Adaptations to Adipose Tissue Thermogenesis. Front. Endocrinol..

[15] Nirengi S., Stanford K. (2023). Brown adipose tissue and aging: A potential role for exercise. Exp. Gerontol..

[16] Esteves J.V., Stanford K.I. (2024). Exercise as a tool to mitigate metabolic disease. Am. J. Physiol. Cell Physiol..

[17] Rodríguez E., Monjo M., Rodríguez-Cuenca S., Pujol E., Amengual B., Roca P., Palou A. (2001). Sexual dimorphism in the adrenergic control of rat brown adipose tissue response to overfeeding. Pflug. Arch. Eur. J. Physiol..

[18] Takabe K., Kim R.H., Allegood J.C., Mitra P., Ramachandran S., Nagahashi M., Harikumar K.B., Hait N.C., Milstien S., Spiegel S. (2010). Estradiol Induces Export of Sphingosine 1-Phosphate from Breast Cancer Cells via ABCC1 and ABCG2. J. Biol. Chem..

[19] Guo S., Yu Y., Zhang N., Cui Y., Zhai L., Li H., Zhang Y., Li F., Kan Y., Qin S. (2014). Higher level of plasma bioactive molecule sphingosine 1-phosphate in women is associated with estrogen. Biochim. Biophys. Acta (BBA)—Mol. Cell Biol. Lipids.

[20] Bengtsson T., Cannon B., Nedergaard J. (2000). Differential adrenergic regulation of the gene expression of the beta-adrenoceptor subtypes beta1, beta2 and beta3 in brown adipocytes. Biochem. J..

[21] Bronnikov G., Bengtsson T., Kramarova L., Golozoubova V., Cannon B., Nedergaard J. (1999). beta1 to beta3 switch in control of cyclic adenosine monophosphate during brown adipocyte development explains distinct beta-adrenoceptor subtype mediation of proliferation and differentiation. Endocrinology.

[22] Bronnikov G., Houstek J., Nedergaard J. (1992). Beta-adrenergic, cAMP-mediated stimulation of proliferation of brown fat cells in primary culture. Mediation via beta 1 but not via beta 3 adrenoceptors. J. Biol. Chem..

[23] Pitman M.R., Lewis A.C., Davies L.T., Moretti P.A.B., Anderson D., Creek D.J., Powell J.A., Pitson S.M. (2022). The sphingosine 1-phosphate receptor 2/4 antagonist JTE-013 elicits off-target effects on sphingolipid metabolism. Sci. Rep..

[24] Lessard S.J., Rivas D.A., Alves-Wagner A.B., Hirshman M.F., Gallagher I.J., Constantin-Teodosiu D., Atkins R., Greenhaff P.L., Qi N.R., Gustafsson T. (2013). Resistance to aerobic exercise training causes metabolic dysfunction and reveals novel exercise-regulated signaling networks. Diabetes.

[25] Raun S.H., Pham T.C.P., Viggars M.R., Ato S., Takagaki Y., Yan Z., De Bock K., Hornberger T.A., Richter E.A., Auwerx J. (2026). How to train your rodent: Recommendations for the preclinical study of exercise-induced benefits in metabolic research. Cell Metab..

[26] Livak K.J., Schmittgen T.D. (2001). Analysis of relative gene expression data using real-time quantitative PCR and the 2(-Delta Delta C(T)) Method. Methods.

